# Accessing the Lived Experiences of Children with Illness in Sweden for Health Care Research

**DOI:** 10.3390/children11121477

**Published:** 2024-12-01

**Authors:** Laura Darcy, Åsa Israelsson-Skogsberg, Ida Kleye, Katarina Karlsson

**Affiliations:** 1 Faculty of Caring Science, Work Life and Social Welfare, University of Borås, 50190 Borås, Sweden; asa.israelsson-skogsberg@hb.se (Å.I.-S.); katarina.karlsson@hb.se (K.K.); 2 Department of Health Sciences, University West, 46186 Trollhättan, Sweden; ida.kleye@hv.se

**Keywords:** children, health research, lived experiences, living with illness, qualitative approaches, children’s rights

## Abstract

Background: Children are a relatively marginalized group when doing research in the context of illness, young children particularly so. This even though children can and should contribute their point of view in providing evidence-based care. This article contributes the experiences of Nurse Researchers in gathering research data in Sweden on the lived experiences of children undergoing needle-related medical procedures, living with home mechanical ventilation or undergoing treatment for cancer. Methods: Nine published articles from four unique Swedish PhD dissertations in Caring Science formed the basis for the present paper where various open and critical qualitative approaches for gathering data were used. Results: Accessing and interpreting the lived experiences of ill children in healthcare research presents methodological, ethical, and cultural challenges. As with health care contexts in other countries, capturing the ill child’s experiences in Sweden requires several different methods. Health researchers in Sweden must navigate a complex landscape of communication barriers, emotional and psychological challenges, and structural issues within the healthcare system to effectively access and understand the lived experiences of children. Conclusions: This paper adds to the knowledge base of research with a focus on gathering the experiences of children with illness within a Swedish health care context. These insights underscore the importance for all researchers of using child-friendly, inclusive methods to understand children’s lived experiences thus ensuring their voices are heard and respected in health research. Children’s and families’ inability to speak the native language of a country cannot be a hinder for inclusion, but rather be encouraged.

## 1. Introduction

Children are a relatively marginalized group when doing research in the context of illness, young children particularly so. Conducting research with children and observing them or encouraging them to talk about their experiences of healthcare is a very important aspect in providing evidence-based care. The healthcare literature has traditionally focused on older children, adolescents’, parents and staff perspectives and proxy descriptions., Children have the right and the ability to contribute unique information about their own experiences. Health care and parental contributions can be helpful but cannot replace the child’s own experiences [1,2]. Gathering data from children has historically been a neglected area but there is an emerging and rich literature om children’s voices and participation in research. Furthermore, children have the right by law to have their voices heard in healthcare, particularly in light of United Nations charter on the rights of the child [3]. which is Swedish law since 2020.

Research with children raises special considerations, which require a heightened sensitivity from the researcher. Children are not little adults; rather they perceive and understand the world in a different way than the adults. They are not even a homogenous group as they have lots of differences as for example age, gender, upbringing, culture and what they favour [4].

Children have sufficient mental ability, cognition and awareness of concepts from as young as three years of age, to enable them to participate and describe their experiences- just not in the same way as adults [5]. Young children’s understanding of illness and health are a function of experiences and understanding, so their understanding is dependent on their previous experiences and everyday life environment, and not just their cognitive abilities [6]. They lack experience, not intelligence, and awareness of individual children’s experiences and developmental level are important in developing and using interview techniques [7]. Directly talking to children about their illness experiences reveals a blend of reality and imagination. Children often express contrasting emotions such as fear and confidence, or sadness and fun, highlighting the complex nature of their experiences. Young children can articulate their experiences in interviews, if the interview questions are developmentally appropriate [8].

Some other methodological approaches that enable capturing children’s perspectives include participatory observation, where researchers interact with participants while simultaneously observing the situation [9]. Video observation can be used to document children’s verbal and non-verbal behavior permanently. It helps capture contextual details and allows for repeated viewing of the procedure [10]. Another approach is taking field notes continuously during data collection [11]. These notes can complement observations and interviews by recording what was not captured through video observation.

The literature highlights important aspects that help adults access the child’s world such as the prominent place of play and imagination and Visual Methods. Play is a crucial tool for children to cope with and make sense of their illnesses. Health Play Specialists (HPS) use playful methods to communicate effectively with children, helping them understand their conditions and procedures. This approach not only aids in communication but also supports the child’s emotional well-being [11]. Techniques like drawing, writing, and photo elicitation are effective in capturing children’s perspectives. These methods empower children by allowing them to express their experiences in their own terms, thus reducing power imbalances between researchers and young participants [10]. Methods like the “Draw and Write” technique and informal interviews have proven useful in accessing children’s perspectives [11]. One way of reaching the child’s experiences is by using Lifeworld interviews as involve a phenomenon-oriented and reflective dialogue [12]. To obtain varied data, detailed descriptions of the phenomena being studied are required. During the interview, participants are encouraged to reflect on the phenomenon, such as play.

Research with children is an increasing area of focus in recognition of children’s capacity to use their voice to share their lived experiences. Research gathering the experiences of ill children themselves is limited and therefore this paper adds to the knowledge base of research with a focus on gathering the experiences of children within a Swedish health context. Additionally, the paper discusses gathering data from young children (from 3-years-old) rather than focusing on older children only. The aim of this paper is to share diverse experiences and knowledge gained from collecting research data on the lived experiences of children with cancer, with home mechanical ventilation or undergoing needle-related medical procedures in Sweden.

## 2. Materials and Methods

Nine published studies from four unique Swedish PhD dissertations (A-D) in Caring Science from 2015–2023, formed the basis for the present paper where various open and critical qualitative approaches for gathering data were used (see Table 1). Data for the studies presented in this paper were gathered using qualitative methodologies through interviews, video filmed interactions, observations or with inspiration from photovoice. Qualitative methods offer the best means of gathering meaningful data from children [13]. Qualitative design offers a flexible methodology, which enhances sensitivity, thus empowering the child’s t and giving the researcher access to the child’s world of meaning and belief [14].

**Table 1:** An overview of all doctoral dissertations and included articles in the present study.

Doctoral Dissertations		Included Articles	Methods	Participants
A	Child centered care during needle procedures: The importance of intercepting and meeting children’s fear and pain.	Kleye, I., Hedén, L., Karlsson., K, Sundler, A., & Darcy, L. (2020). Children’s individual voices are required for adequate management of fear and pain during hospital care and treatment. Scandinavian Journal of Caring Science. doi:10.1111/scs.12865. [15]	Interviews and Video observations	13 children aged 4–12 years
		Kleye I, Sundler AJ, Darcy L, Karlsson K, Hedén L. Children’s communication of emotional cues and concerns during a preoperative needle procedure. Patient Educ Couns. 2022 Jun;105(6):1518–1523. doi: 10.1016/j.pec.2021.09.035 [16]		26 children aged 4–12 years
B	Children living with Home Mechanical Ventilation: The everyday life experiences of the children, their siblings, parents and personal care assistants.	Israelsson-Skogsberg, A., Heden, L., Lindahl, B., & Laakso, K. (2018). ‘I’m almost never sick’: Everyday life experiences of children and young people with home mechanical ventilation. Journal of Child Health Care, 22 (1) 6–18 doi:10.1177/1367493517749328. [17]	Interviews inspired by Photovoice methodology	13 children aged 7–12 years
		Israelsson-Skogsberg, A., Markström, A., Laakso, K., Heden, L., & Lindal, B. (2019). Siblings’ lived experiences of having a brother or sister with Home Mechanical Ventilation—A phenomenological hermeneutical study. Journal of Family Nursing 25 (3), 469–492. doi:10.1177/1074840719863916. [18]		
C	“I’m afraid, I want my mommy”: Younger children’s, parents’, and nurses’ lived experiences of needle procedures in health care.	Karlsson, K., Dalheim Englund, A., Enskär, K., Nyström, M., & Rydström, I. (2016a). Experiencing Support During Needle-Related Medical Procedures: A Hermeneutic Study With Young Children (3–7 Years). Journal of Pediatric Nursing, 31(6), 667–677. doi:10.1016/j.pedn.2016.06.004. [19]	Lifeworld interviews and filmed interactions	21 children aged 3–7 years
		Karlsson, K., Rydström, I., Nyström, M., Enskär, K., & Dalheim Englund, A. C. (2016b). Consequences of Needle-Related Medical Procedures: A Hermeneutic Study With Young Children (3–7 Years). Journal of Pediatric Nursing, 31(2), e109–e118. doi.10.1016/j.pedn.2015.09.008. [20]		
D	The everyday life of young children through their cancer trajectory.	Darcy, L., Knutsson, S., Huus, K. and Enskar, K., 2014. The everyday life of the young child shortly after receiving a cancer diagnosis, from both children’s and parent’s perspectives. Cancer nursing, 37(6), pp. 445–456. [21]	Interviews at 3–9 weeks, and 6, 12, 18 and 36 months post diagnosis	13 Children aged 3–6 years
		Darcy, L., Björk, M., Enskär, K. and Knutsson, S., 2014. The process of striving for an ordinary, everyday life, in young children living with cancer, at six months and one year post diagnosis. European Journal of Oncology Nursing, 18(6), pp. 605–612. [22]		
		Darcy, L., Enskär, K. and Björk, M., 2019. Young children’s experiences of living an everyday life with cancer–A three year interview study. European Journal of Oncology Nursing, 39, pp. 1–9. [23]		

Children aged 4–12 participated in two different studies that were part of one of the dissertations (A) (Table 1: [15,16]). One of the studies was conducted with 13 children who all had experience of being cared for in hospital for at least two weeks. The purpose of this study was to gather knowledge and experiences from children about which strategies they use to manage their fear and pain in connection with difficult treatment. To facilitate the interviews, a Polaroid camera was used. The children took photographs when they walked around with the researcher or remained inside the patient room. In this way, the interaction with the researcher was strengthened and the interview led to a conversation with space for the child’s voice. Both objects and places that had caused them fear or pain were photographed by the children. Furthermore, the researcher conducted video observations when a total of 26 children were to undergo a needle procedure. The purpose of these studies was to study the communication of unpleasant emotions between children and nurses during needle procedures. Through video recording, careful analysis of the children’s communication could subsequently be carried out. The video recordings enabled the children’s non-verbal communication to be expressed and given a voice.

In another dissertation (B), 13 participants aged 7–12 years of age were interviewed (Table 1: [17,18]). Participants were six children with home mechanical ventilation (HMV), and seven siblings to a brother or sister with HMV. A qualitative research methodology in terms of interviews combined with inspiration from photovoice methodology [24] were suitable to stimulate the participants’ own unique stories about their everyday life. Some of the participants with HMV had a very weak voice and impaired communication skills due to a muscle disease, and they suffered from shortness of breath. Consequently, a carefully designed interview approach characterised by both sensitivity and flexibility was needed. Interviews were conducted with children with HMV and their siblings with inspiration from photovoice methodology [25], which combined photography with storytelling, allowing children to express their thoughts, experiences, and perspectives visually. The photographs became a suitable way to increase the child’s level of confidence and establish a relationship between the child and researcher.

In a third dissertation (C), data were gathered from four different paediatric healthcare settings with 21 children aged 3–7 years undergoing a needle-related medical procedure (NRMP) (Table 1: [19,20]). The gender distribution of the children was 11 girls and 10 boys. The types of NRMPs included capillary blood samples, venous blood samples, intravenous cannula insertion (IV), needle insertion into a central venous port, injections into the joints, and skin tests for allergies. The children received pharmacological aids according to the applicable protocols within each unit. These studies were inspired by a lifeworld hermeneutic approach [12]. The meaning-oriented interviews consisted of a phenomenon-oriented and reflective dialogue between the researcher and the child. Interviews were conducted with children after the needle-related medical procedure was completed. The data collection consisted of participant observations and interviews, documented with video-recorded observations, field notes, and audio recordings. A lifeworld hermeneutic approach was used, with meaning-oriented interviews consisting of a phenomenon-oriented and reflective dialogue [12] between the researcher and the child undergoing a needle-related procedure.

Data for a fourth dissertation (D) involved interviewing children aged three to six years of age, and the parents of all children from one year of age, at five time points over a three-year period (Table 1: [21,22,23]). Consecutive inclusion of children being treated at a paediatric oncology center in the West of Sweden was made. Longitudinal studies where the researcher and participants meet at several time-points over 3 years offered another approach to data gathering. This longitudinal, inductive study was part of a larger project exploring young children’s experiences of how cancer affects their everyday health and functioning, over a three-year period from diagnosis. Data was collected at several time-points and inspired by the framework of the World Health Organisation’s International Classification of Health, Functioning and Disability [26]. The focus was on the child’s experiences of their changing body and functioning, what they did every day and with whom and what environmental support was given or deemed necessary to do the things they wished to do. The continuous adjustments children had to make to their lives due to the prolonged nature of childhood cancer, was captured using several data collections. An inductive approach with Semi-structured interview questions were the most appropriate way to access the stories of everyday lives of young children with cancer [13].

### Ethical Considerations of Research with Children

Ethical considerations that are made when doing research with adult research subjects can and must be applied to children too [27]. All four dissertations (A–D) were carried out in accordance with the Helsinki Declaration [28], data confidentiality were safeguarded in accordance with Swedish Law in both the original dissertations and the present study [29]. The four ethical principles of Respect for Autonomy, Beneficence, Maleficence and Justice were met: children had the right to make their own choices, the researcher acted with the best interest of the children in mind, no harm should be done and fairness and equality among individual children was enacted [28]. Children received both written and verbal information prior to inclusion and verbal information prior to each interview. The text was written in an easily understandable language and pictures were added to enhance understanding, including a picture of the researcher. The parents received more detailed written and verbal information. Time was given to both adults and children to ask questions before consenting. Both the child’s parents gave their informed consent for their own and the child’s participation. Children themselves assented to participate in the 11 included studies that form the basis of the present paper [30] rather than consented, as they are minors and therefore unable to give legal consent [31,32].

## 3. Results

Based on the nine included articles, the results section presents the main areas that summarize the experiences of gathering research data with children with illness in a Swedish health care context. The main areas have been created by identifying the common shared experience of the nine articles.

### 3.1. Settings for Interaction with the Child

When children and families themselves decided on the setting for the interviews, they often opted to meet in the participant’s own home. This facilitated the child’s unique needs regarding medical conditions, requirements and way of communicating. Creating a safe and secure space was important to enable the participants to speak openly. Interactions with children in their home were all different in its character; some participants wanted to be in different areas in the house while talking and showing important things, some wanted to lie down on the living room floor or to sit on the floor of their bedroom. Being allowed into the family home meant being invited to witness their everyday lives. Friends and relatives arrived, siblings cried, and dogs were barking. There was often a lot of noise and activity going on during the interview, creating a very dynamic and exciting situation.

Parents were present during interactions conducted in healthcare settings, sometimes with siblings or a relative. If both parents were present, they both listened and participated but with the child in focus. Staff interrupted the interview situations when something needed to be done with the child, sometimes leading to stress for the parents and the interviewer. All of this together sometimes made the interview situation seem cluttered, but under the circumstances, it appeared to be the only option. It was challenging to make the technical equipment work, such as determining where and how best to place the cameras in the room to get enough light and sound.

### 3.2. The Use of Play and Props

Various toys and tools were used as younger children may have difficulty talking about their experiences in answer to a direct question. The child was asked to look in a bag that was presented to them as a “secret bag”. In the bag there was medical equipment, soft toys and dolls. For example, when the child played with the doll it could demonstrate the child’s experiences during procedures, as part of the recorded interview. The doll indirectly “asked questions” to the child about their experiences. The child could also choose a prop to help them mirror their experiences, such as sad and smiley faces. Some children preferred to look in photo albums with pictures of other children going through similar procedures or to draw pictures that illustrate the child’s experiences or indeed through free play. Not all children wanted to play with dolls or soft toys, rather they were offered as alternatives.

### 3.3. Using Cameras and Photos

In some of the studies, a polaroid camera was used to facilitate the interaction between children and researchers. The children were tasked with photographing medical equipment, other things or places that caused them experiences of fear and pain. Based on this assignment, the children and the researcher had to walk around the hospital together, which contributed to the fact that the children and the researcher had something in common. This commonality contributed to the children conducting the interviews in a more open and natural way. For some children, the interview took place in connection with the taking of the photos, while other children wanted to sit down and look at the taken photos in order to then talk about them. The photographs were not analysed.

In other studies, children were invited to photograph various things using the camera in their mobile phone that they regarded as essential in their everyday life, prior to meeting the researcher. In some cases, the researcher and child strolled around, the child took photos, showing favourite toys or medical equipment and told stories about the photographed objects. The images and stories related to them included favorite friends, domestic pets and bottles of hand disinfectant. The photos were used to facilitate conversation by giving the participants the opportunity to initiate conversations by talking about their own photos—these photos were not analysed separately. Some key advantages were that the photographs engaged the children more actively in the interview process and it served as conversation starters, helping children articulate their thoughts and feelings more effectively during the interview. Younger children’s stories and conversations were very concrete such as distinct memories of situations, places and toys. The photographs helped them to explain and develop their stories. Older children described more abstract phenomena such as emotions and empathy. Overall, photos provided a creative and inclusive approach to gathering insights from the participants that allowed us as researchers to gain a deeper understanding of their perspectives.

### 3.4. Filmning and Observing

One way to capture the child´s facial expressions, gestures and postures during interview was by observation through video-recording the whole situation, as one researcher did. During the interview, the child watched the film/video-recorded observation from the needle procedure, and answered questions posed by the interviewer. Interviews were complimented with documented with field notes, which were conducted in a discreet and limited manner and notes that are more detailed were made when the needle procedure or interview was completed.

Using video recordings captured children’s communication, verbal and non-verbal, during or in connection with care and treatment. By, in this case, placing a video-camera at the foot of the child’s bed as in one study, the researcher was able to capture the verbal and non-verbal communication with healthcare professionals.

As an interviewer, it was challenging to ensure focus remained on the child being interviewed rather than the technical equipment. The equipment could distract the children, who became more interested in the video camera, for example, than in answering interview questions. Allowing the children to look at and examine the equipment and ask questions reduced their interest in it.

### 3.5. Verbal Interaction with Children

Interviewing younger children whose ability to express oneself may be limited can be challenging. Some of the children had a reduced physical ability to speak related to a severe muscle disease. Many children were afraid of a procedure they were to undertake. A preparatory phase preceded the interviews with the purpose of familiarising the child with the situation. This included small talk and play, depending how the child steered the situation When the child seemed to feel comfortable with the situation, the conversation was directed against the initial question, which varied slightly depending on the child’s age, level of maturity, experience, mood and current condition. A progression in the children’s ability to answer questions was seen by researchers that met with the child several times. at their second and sequential interviews. As they came to terms with a new everyday life, children learned how to talk about the focus in the interview. They also matured cognitively over time.

As Sweden is a multicultural society, researchers made it possible for children and parents who do not speak the language to be included in studies. This was made possible by using professional interpreters. The interpreters were used both to try to ensure that parents and children understood the meaning of participating in the research and to translate what the healthcare professionals wanted to convey verbally to the child. The interpreters stood behind the researcher and directly translated what was mediated between the researcher and children and parents.

### 3.6. The Availability of Parents

An important resource in accessing ill children was the availability of parents and researchers deemed it inappropriate and unethical to separate child and parent in the research situation. All the children participating in the studies in the present paper decided themselves if they wanted a parent nearby during the interviews. Younger participants wanted to have a parent beside them or at least nearby, whereas the older participants chose not to. Parents and children spoke out at the same time and finished each other’s sentences when the child’s own voice became fragile and weak, due to e.g. a decrease in muscle strength. In some instances, the parent just sat beside the child without taking part in the conversation, while in other cases they supplemented the child’s story by asking their child questions such as “Do you remember what you told me yesterday” or “How did it feel when we did that?”. The different voices recorded were marked with great accuracy in the transcribed material. This was a meticulous process aiming to ensure that the children’s own voices comprised the main data. Parental contributions were rigorously kept to their child’s experiences, complementing the child’s voice rather than being the child’s voice.

### 3.7. Designated Nurses as Gatekeepers

For the published papers this study is based on, designated nurses took the first contact with the parents and their children to give information about the study. If the parents and the child showed interest about the study, the parents gave their written consent for their names and contact information to be forwarded to the researcher, to get further information about the study. We have not encountered that with the designated nurses in our studies, as we have had good contact with them and informed them well about their responsibility. However, the nurses on the ward have sometimes tried to act as gatekeeper for the child and its parents. For example, there have been times when the researcher had decided an interview time with the child and the parent. At arrival to the ward, it then happened that the nurse on the ward said that the family had a very rough time and that she thought it was not suitable to perform an interview. However, it was in none of these times that the parents and children said no, instead they said that they really wished to talk with the interviewer about what was the aim of the interview. This highlights that it is of importance to let the research subjects decided themselves, as they can do that even if they live through an arduous time. In our experience, it is of importance that designated nurses understand the importance and relevance of nursing research and be objective and empathetic. The authors of the present paper recommend returning to the clinics involved in caring for children in studies and presented results, sometimes several times.

### 3.8. Ethical Dilemas

Researchers faced challenges such as ensuring the child’s comfort and trust, and the ethical considerations of involving children in research. In the case of younger children, obtaining informed consent can be challenging, as they may have difficulties understanding and contextualizing events. However, as much as possible, younger children should give their consent (assent) to participate in the research. Some of our experiences as researchers were that scheduled meetings were cancelled several times because of various infections and illness. In some studies, the participants were from a relatively small group of children, with rare diseases. Strategies to protect identification were very important, which included careful selection of elaborate examples and excluding detailed information. This was very important to explain for the participants. Dilemmas occurred which raised ethical concerns, but which has nothing to do with the study e.g. the need for extra support in the home or lack of appropriate pain relief in a care situation. The dilemmas experienced by the researcher in a research setting were discussed with consultant experts for guidance, without revealing the identities of those involved.

## 4. Discussion

The results of the present study reveal that opportunities and challenges to accessing lived experiences of ill children in Sweden are similar to international research. We found that children can and should be included in research as a valid way of ensuring their experiences are recognised. However, accessing and interpreting the lived experiences of ill children in healthcare research presents methodological, ethical, and cultural challenges. Accessing the lived experiences of ill children in Sweden reveals the importance of family dynamics, the need for supportive healthcare structures, and the critical role of trust and communication. As in line with international research in health care, a whole range of methods need to be employed to fully capture the ill child’s experiences in Sweden.

Several research challenges involving children were revealed. The relatively small numbers of children with serious medical difficulties and special ethical and regulatory protection, were challenges revealed. The complexities of parental involvement, specific child friendly adaptations in procedures and settings and the need for developmentally appropriate outcome measures were other also raised as challenges. Obtaining permissions from multiple ethics committees and healthcare institutions can be time-consuming and complex, often leading to delays in research initiation. Gaining consent from both parents and children adds another layer of complexity, especially in ensuring that children are adequately informed and willing participants [33]. When researching with children in health care, difficulties with access to participants can occur. Research projects that ask children about aspects of their family lives lead to ethical tensions, and methodological strategies are needed to mitigate these challenges [34]. Swedish research has pointed out that leisure time is very valuable as families often carry an excessive burden of care involving many care activities [35]. Children are already a vulnerable group in care and research and various treatments and disabilities may make them even more vulnerable. Another challenge we met in our studies were long distances to travel to meet the children and their families.

Meeting the child in their home or healthcare setting was a privilege and gave unique insight to their everyday life and lived experiences. We also encountered challenges when meeting the child in both settings with the presence and interaction of siblings, friends, relatives and pets. This concurs with previous literature on the chaos and complexity of interviewing children and families in their own homes. They describe how dealing with perceived chaos is challenging, but because children communicate with others through their environment, capturing the situation is part of the complexity of qualitative enquiry [36]. After completing cancer treatment, families experience a mix of relief and ongoing strain. Parents often feel they are in uncharted territory and miss the security provided by hospital settings. Previously sick children may feel a loss of parental concern post-treatment, while siblings might receive more attention, leading to complex family dynamics [37]. 

Structural Issues for immigrant families exist in Sweden such as language and cultural issues and challenges in navigating the healthcare system. Immigrant families often face difficulties in communication due to language barriers and cultural differences, which can hinder effective data collection and understanding of their experiences. They also report difficulties in navigating the Swedish healthcare system, which can complicate researchers’ efforts to engage with these families and understand their experiences [38,39]. Our studies show using interpreters to access the experiences of children without sufficient knowledge of the native language of the country is possible and should be encouraged. By opening up to and enabling children of all backgrounds to participate in research, research results increase in credibility, validity, reliability and transferability.

Children in Swedish research emphasize the importance of feeling appreciated, respected, and listened to for their overall health and well-being. These factors are crucial in promoting health literacy among young people [40,41]. Talking to a researcher and sharing the illness story can be comforting and healing [42,43,44]. We found that by using developmentally appropriate props in a safe environment where the child feels secure and leads the way, children were able to articulate their experiences. We concur that children can more easily communicate their experience when using images and toys in health care research. Toys and images can also positively affect the power imbalance that may exist between child and adult researcher [45].

A concern about asking children to participate with video recordings is that many children will refuse, because the camera will make them feel uncomfortable. But in today’s digital society with children growing up with cameras in their pockets, this has not been a problem. Instead, video recordings have produced results that would have been difficult to capture by other data collection methods.

Contact with children and their families in health care research needs to be initiated by an independent third party in line with ethical guidelines [28]. Earlier research [46] has pointed out that it is important to have a good relationship with the healthcare setting. Previously, researchers have experienced those designated persons such as nurses as gatekeepers, as they have described as trying to protect the child against research. Literature has described how this “protection” role could effectively disempower the research subjects from researching their own decisions. We found it of vital importance to gain the trust of designated healthcare staff and show that we as researchers were honest, reliable and communicate well. Confidence improves with peer support and collaboration with pediatric clinics. Building trustful alliances between healthcare providers and families is crucial. Lack of trust can lead to increased anxiety and a sense of overwhelming responsibility among parents [47].

Ethical considerations are vital when accessing the lived experiences of sick children in healthcare research [34]. Ethical dilemmas occurred in some situations we met in our studies, both at home and in hospital settings. This is not unusual when a professional researcher is also a caring professional. The awareness of possible differences in research contra clinical roles should be an important aspect of training for research students [4]. Another challenging situation can be associated with the role of power a researcher in health care possesses in the research situation. It is therefore important to reflect on the fact that power always carries a potential risk for abuse. Meeting with the child several times, as our studies showed, builds rapport between the researcher and the participant over time [43]. Researchers should be aware that gathering data from children in longitudinal studies can have an impact on them and their answers. Children with health challenges often mature quickly due to their experiences and time spent with adults. This may positively influence their understanding of the research question and data, even at a very young age [48].

According to international literature, involving children in research often benefits from a family-centered approach, where the experiences of the entire family, including parents and siblings, are considered. This method acknowledges the relational dynamics and responsibilities within families, making data collection safer and more comprehensive [47]. The availability of parents makes children feel safe and secure, which helps them express their needs [49]. The child’s attachment behavior is activated at times of stress and can be eased by the presence of their “secure base” [42]. Gathering verbal information from a very young child is challenging, and therefore parent’s perspectives on the child’s experiences can also contribute to our understanding of the child’s world [50]. The presence of parents, as experts on their child, was in some cases necessary for the child’s voice to be heard. However, as researchers, we were aware of impacts of parental presence in the interview situation, comforting but perhaps restricting the ability to speak freely. Parents expressed challenges in being an expert on the ill child, as the context, illness, treatment and child’s behaviour were new to them. Our experiences align with the research that children, even young children, are the best sources of information about themselves [7].

In order to be able to meet each child’s unique competence and experience, it is required that researchers have knowledge and insight into the difference between an adult child’s perspective and the child’s own perspective. An adult child-perspective means that an adult tries to understand the child’s experiences and actions based on the child’s view of the world. The children’s perspective is a fundamental prerequisite for researchers to be able to carry out research methods that include and capture children’s natural participation [2]. As a result of the fact that adults can never fully take the child’s perspective, children must be studied based on different methods to get closer to their perspective. The current study highlights and demonstrates various qualitative methods for data collection with children, but quantitative methods can also give children a voice. Examples of this are, among other things, assessment instruments that are common when assessing, among other things, fear and pain. With this study we propose a child-centered approach rather than a traditional family centered approach where the child’s different ways of communicating such as verbal and non-verbal expressions, perceptions and understandings are in focus and where we use several methods that are adapted to their developmental level.

To fully explore children’s perspectives, researchers need to focus on the child. A prerequisite for involving children in the process is that the child/children are regarded as independent actors and that they are given the opportunity to have their own space [51]. A group cannot be excluded from research because the Challenging data collection cannot be a premise for denying research with children. All children should be included in research independent of age, ability to answer or mother tongue. Adults hold the power, but children have the right to be researched on and with [30]. It would be both unethical and unscientific to exclude those that cannot make their own decision [52]. To achieve best access to the world of the child, their participation in research should start with planning the study design. The present study is based on nine articles, all of which were conducted in a Swedish context, which could affect the transferability of the research findings to other contexts or groups [53]. But a well-detailed description of the context allows readers and other researchers to assess whether the findings can be meaningful for groups outside the studied sample. Such descriptions are essential to make the re-search useful for others working with similar populations [53]. All nine articles in this present study [15,16,17,18,19,20,21,22,23] are carried out in well experienced research groups considering research with children and qualitative methods which is regarded as a strength of the present study and promote trustworthiness [53].

## 5. Conclusions

This article has described experiences of gathering qualitative research data from young and ill children in Sweden. Health researchers in Sweden must navigate a complex landscape of communication barriers, emotional and psychological challenges, and structural issues within the healthcare system to effectively access and understand the lived experiences of children. We have shown that research with very young children is possible using a range of methods. Children’s and families’ inability to speak the native language of a country cannot be a hinder for inclusion, but rather be encouraged. Our addition to the literature is a Swedish children’s rights perspective and a proposal for a more child-centered care in researching with this vulnerable but capable population of health care users. Our hope is that this information will continue to empower researchers in accessing the young, ill child’s experiences and on the child’s terms both in Sweden and beyond. Further research could focus on the experiences of young, ill children in other countries and the terminology used for children with various disabilities. All children have the right to be heard, and researchers have the appropriate tools to do so.

## Data Availability

No new data were created or analysed in this study. Data sharing is not applicable to this article. The original contributions presented in this study are included in the article.
